# Supplementary material for: Motor innervation directs the correct development of the mouse sympathetic nervous system
Source: Nat Commun. 2024 Aug 16;15:7065. doi: 10.1038/s41467-024-51290-0 (PMC11329663; doi:10.1038/s41467-024-51290-0)
Supplement: Supplementary file 1 — Supplementary Information [file 41467_2024_51290_MOESM1_ESM.pdf]

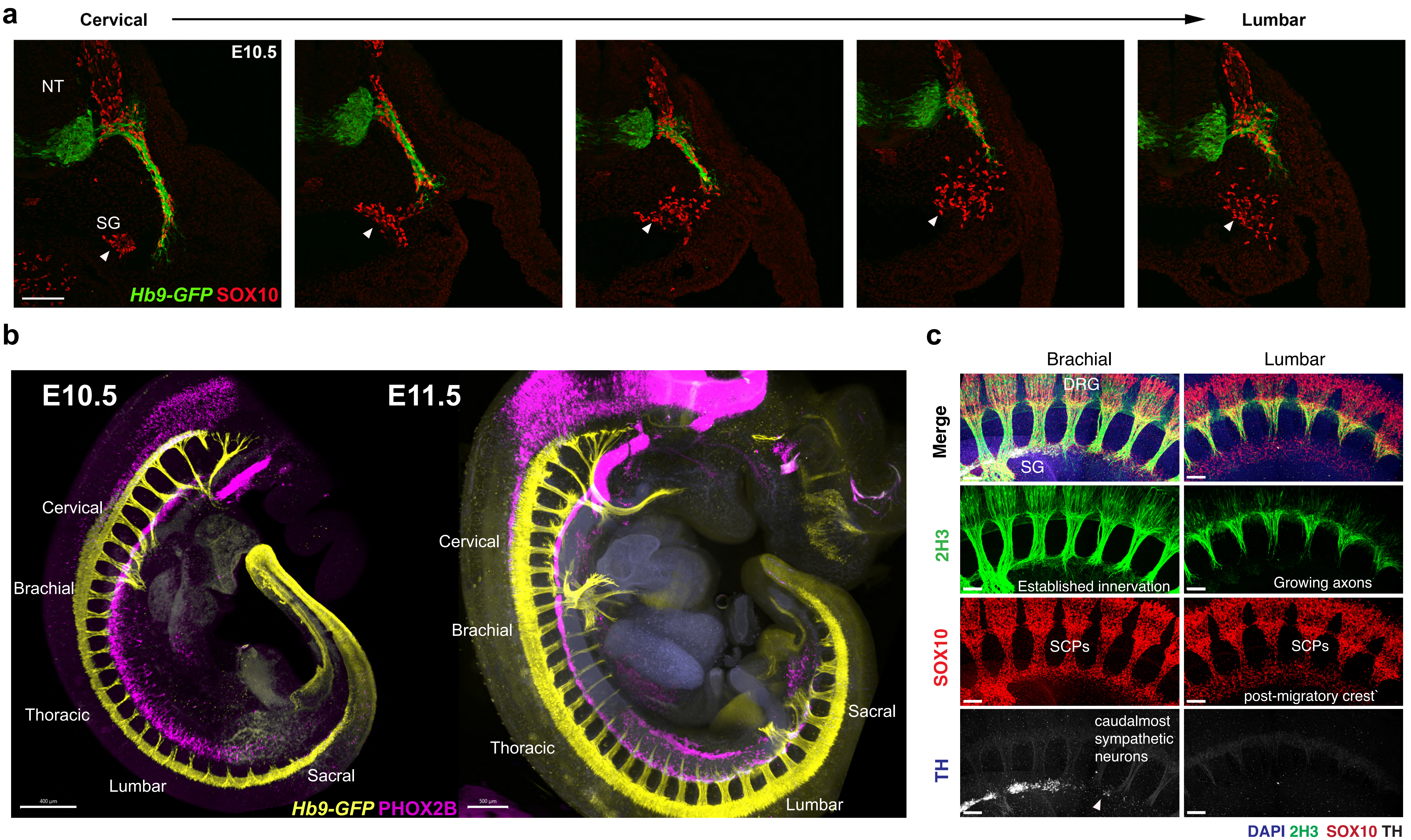
a
Cervical
Lumbar
E10.5
NT
SG
Hb9-GFP SOX10
b
E10.5
E11.5
Cervical
Brachial
Thoracic
Lumbar
Sacral
400 μm
Hb9-GFP PHOX2B
500 μm
Cervical
Brachial
Thoracic
Sacral
Lumbar
c
Brachial
Lumbar
Merge
DRG
SG
2H3
Established innervation
Growing axons
SOX10
SCPs
SCPs
post-migratory crest`
TH
caudalmost
sympathetic
neurons
DAPI 2H3 SOX10 TH

Figure S1: Antero-posterior gradient of motor axon outgrowth and NCC/SCP migration. **(a)** Series of transverse sections from a single E10.5 *Hb9-GFP* embryo ordered from cervical to lumbar segments to recreate a developmental time series. $SOX10^+$ cells (red) progressively associate with motor nerves (GFP, green) as they extend toward the sympathetic ganglia (arrowheads). **(b)** Sagittal view of E10.5 (left) and E11.5 (right) *Hb9-GFP* embryo in wholemount, stained for PHOX2B (magenta) and GFP (yellow). Relevant axial levels are labeled to the left of each embryo. **(c)** Sagittal view of E10.5 embryo wholemount stained for peripheral innervation (2H3), SOX10, and the sympathetic anlagen (TH). At brachial levels (left) peripheral innervation is already well established and the sympathetic anlagen is in contact with sensorimotor fibers, whereas more caudally (right; lumbar), sensorimotor axons have not yet reached the sympathetic anlagen. Note that no TH^+ cells are visible in the presumptive autonomic domain in the lumbar region while sympathetic maturation has already begun at brachial level. All images in (a-c) are representative of 3 embryos. Scale bars: a: 100 µm; b: 400 µm (left), 500 µm (right); c: 80 µm. DRG: dorsal root ganglia; SG: sympathetic ganglia; NCCs: neural crest cells; NT: neural tube; SCPs: Schwann cell precursors.

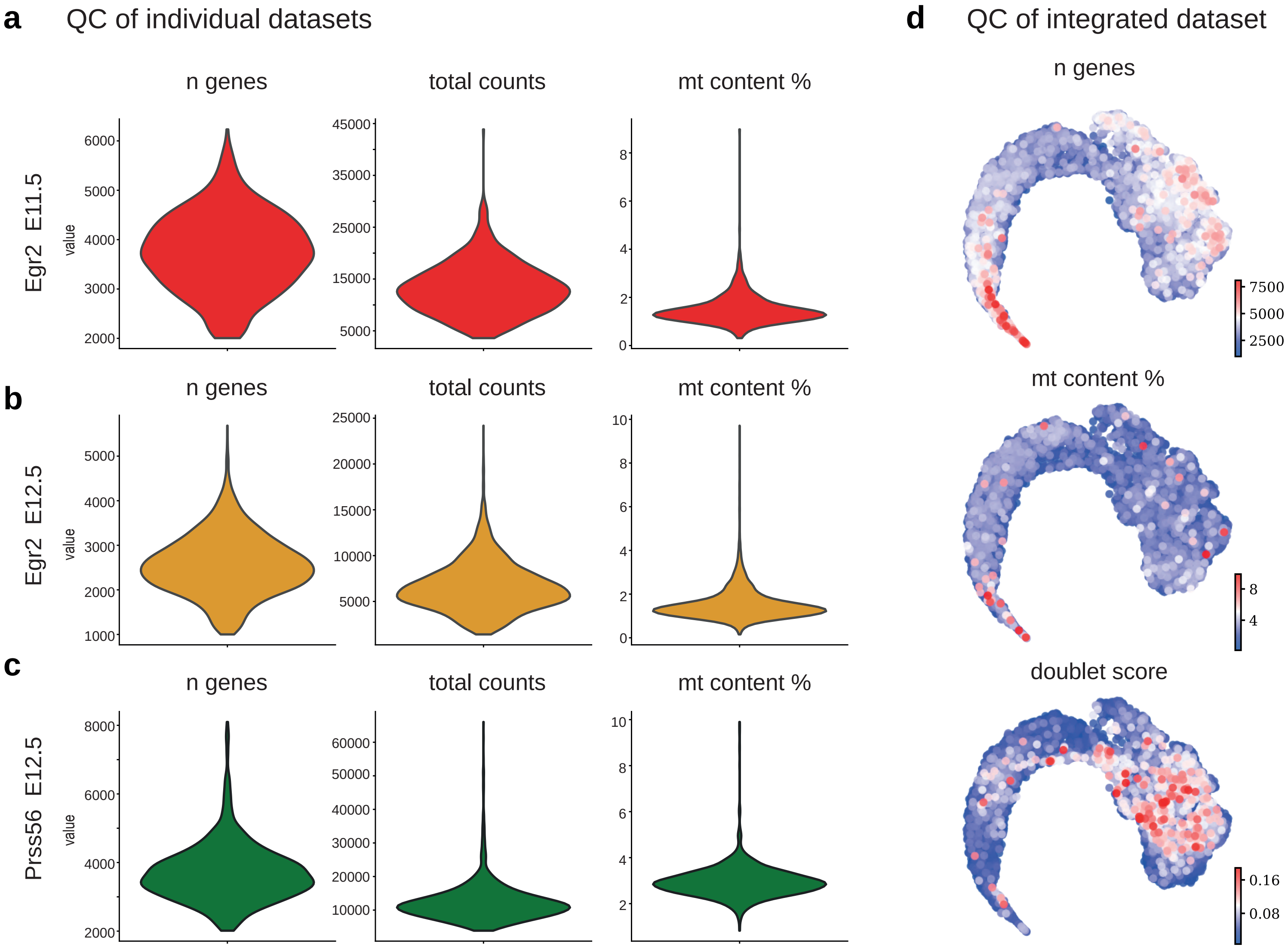
a
QC of individual datasets
n genes
total counts
mt content %
Egr2 E11.5
value
b
Egr2 E12.5
c
Prss56 E12.5
d
QC of integrated dataset
mt content %
doublet score
7500
5000
2500
0.16
0.08

Figure S2: Quality control for the boundary cap tracing scRNAseq experiment. Violin plots showing the number of genes detected per cell (left), the total mRNA counts detected per cell (middle), and percentage of mitochondrial transcripts detected per cell (right) for each dataset. **(a-b)** Quality control for *Egr2-Cre; R26-Tomato* harvested at E11.5 (a) and E12.5 (b). **(c)** Quality control for *Prss56-Cre; R26-Tomato* harvested at E12.5. **(d)** QC metrics number of genes detected, mitochondrial content, and doublet probability (from top to bottom) in integrated dataset. Per condition, traced cells from one litter of embryos were pooled to yield each of the three datasets.

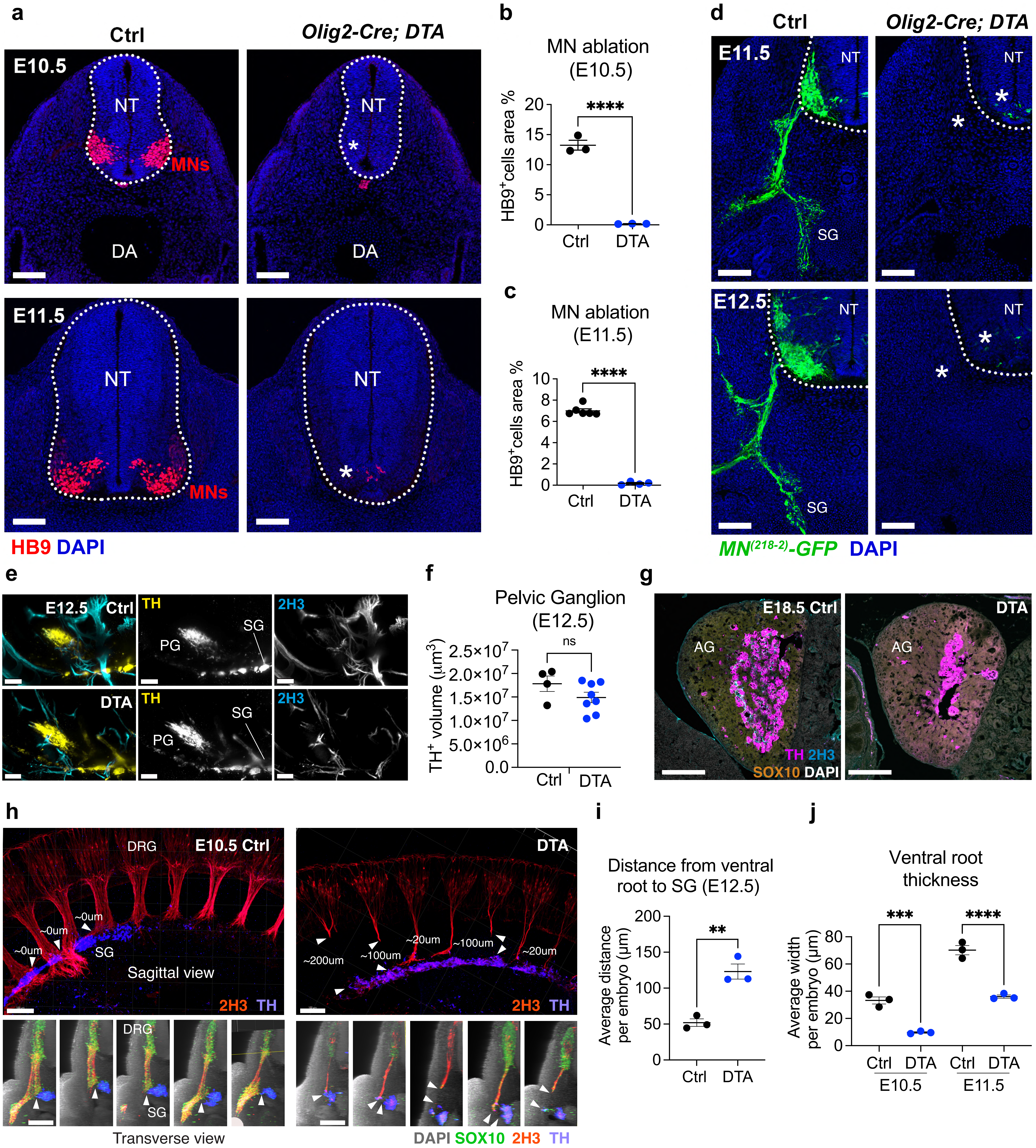

a
Ctrl
Olig2-Cre; DTA
E10.5
NT
MNs
DA
E11.5
HB9 DAPI
b
MN ablation (E10.5)
HB9⁺cells area %
****
Ctrl
DTA
c
MN ablation (E11.5)
d
NT
SG
E12.5
MN(218-2)-GFP DAPI
e
E12.5 Ctrl
TH
2H3
PG
DTA
f
Pelvic Ganglion (E12.5)
TH⁺ volume (μm³)
ns
g
E18.5 Ctrl
AG
TH 2H3
SOX10 DAPI
h
DRG
E10.5 Ctrl
~0um
Sagittal view
~200um
~100um
~20um
Transverse view
DAPI SOX10 2H3 TH
i
Distance from ventral root to SG (E12.5)
Average distance per embryo (μm)
**
j
Ventral root thickness
Average width per embryo (μm)
***
E10.5
E11.5

Figure S3: Efficiency of Diphtheria Toxin A-mediated motor neuron ablation. (a) Transverse sections of the neural tube from E10.5 (top) and E11.5 (bottom) embryos showing complete loss of motor neurons (asterisks) in *Olig2-Cre; DTA* (right). The motor columns are identified by HB9 staining (red) in control littermates (left). **(b, c)** Percentage of $HB9^+$ area within the neural tube of *Olig2-Cre; DTA* and controls at E10.5 (b) and E11.5 (c). Mean ± SEM, Unpaired t test (****) p<0.0001; E10.5: controls n=3, mutants n=3; E11.5: controls n=6, mutants n=4. **(d)** Transverse view of E11.5 (top) and E12.5 (bottom) embryo trunk showing complete loss (asterisks) of motor projections in *Olig2-Cre; DTA* (right). Motor nerves are visualized with $MN^{(218\text{-}2)}$*-GFP* reporter in controls (left). Representative of n=2 control and n=3 mutant E11.5 embryos, and n=2 control and n=4 mutant E12.5 embryos. **(e)** Pelvic ganglia (identified by TH staining, yellow) in E12.5 controls (top) and *Olig2-Cre; DTA* (bottom). 2H3 staining (cyan) labels peripheral nerves. **(f)** Quantification of pelvic ganglion volumes in control and *Olig2-Cre; DTA* embryos. Mean ± SEM, Unpaired t test ns: p=0.1656; controls n=4, mutants n=8. **(g)** Adrenal gland sections from E18.5 *Olig2-Cre; DTA* embryos (right) and control littermates (left) immunostained for TH to identify chromaffin cells (n=3 embryos per genotype). 2H3 staining identifies adrenal medulla innervation, while SOX10 marks satellite glia. **(h)** (Top) Sagittal views of E10.5 *Olig2-Cre; DTA* embryo (right) or control littermate (left) stained for 2H3 and TH. (Bottom) Transversal views of the sympathetic chain and corresponding peripheral innervation at different rostrocaudal levels with 2H3, SOX10, TH and DAPI staining. Arrowheads mark the distance between nerve tip and sympathetic anlagen. **(i)** Average distances between TH^+ sympathetic ganglia and $2H3^+/TH^-$ nerves in E12.5 *Olig2-Cre; DTA* and controls. Mean ± SEM, Unpaired two-sided t test (**) p=0.0038; controls n=3, mutants n=3. **(j)** Average diameter of peripheral nerves in motor-ablated and control trunks, measured in whole mount at E10.5 and E11.5. Mean ± SEM, ordinary one-way ANOVA with Tukey's multiple comparisons test (***) p=0.0003 (****) p<0.0001; controls n=3, mutants n=3. DRG: dorsal root ganglia; NT: neural tube; SG: sympathetic chain ganglia; PG: pelvic ganglion; AG; adrenal gland. Scale bars: a, d: 100 µm; e: 200 µm; g: 200 µm; h: 100 µm.

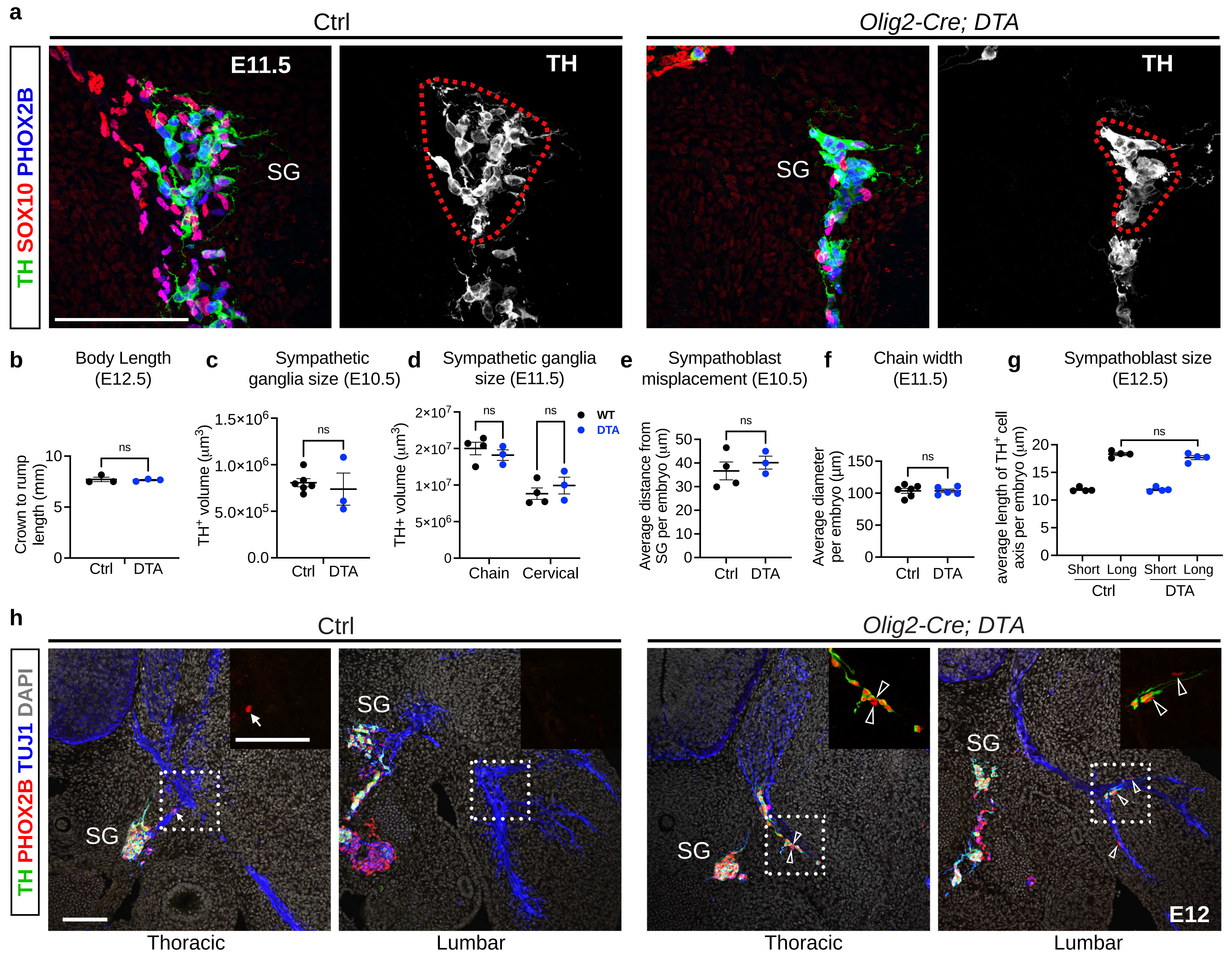

a
Ctrl
Olig2-Cre; DTA
TH SOX10 PHOX2B
E11.5
TH
SG
SG
TH
b
Body Length (E12.5)
Crown to rump length (mm)
ns
Ctrl
DTA
c
Sympathetic ganglia size (E10.5)
TH+ volume (μm3)
ns
Ctrl
DTA
d
Sympathetic ganglia size (E11.5)
TH+ volume (μm3)
ns
ns
WT
DTA
Chain
Cervical
e
Sympathoblast misplacement (E10.5)
Average distance from SG per embryo (μm)
ns
Ctrl
DTA
f
Chain width (E11.5)
Average diameter per embryo (μm)
ns
Ctrl
DTA
g
Sympathoblast size (E12.5)
average length of TH+ cell axis per embryo (μm)
ns
Short
Long
Short
Long
Ctrl
DTA
h
Ctrl
Olig2-Cre; DTA
TH PHOX2B TUJ1 DAPI
SG
SG
SG
SG
E12
Thoracic
Lumbar
Thoracic
Lumbar

Figure S4: Motor nerves prevent ectopic sympathetic differentiation on sensory nerves and control ganglia morphology. (a) Transverse view of E11.5 sympathetic chain ganglia in controls (left) and *Olig2-Cre; DTA* (right) stained for SOX10 (red), PHOX2B (blue) and TH (green). TH staining is also shown separately. The dotted red line identifies sympathetic ganglia. Representative of n=5 embryos per genotype. **(b)** Body length of E12.5 *Olig2-Cre; DTA* and littermate controls from Figure 5. Mean ± SEM, Unpaired two-sided t test, ns: p=0.7817; controls n=3, mutants n=3. **(c)** Volume of TH^+ domains in E10.5 *Olig2-Cre; DTA* and littermate controls. Mean ± SEM, Unpaired t test ns: p=0.6134; controls n=6, mutants n=3. **(d)** Volume of sympathetic chain and cervical ganglia in E11.5 *Olig2-Cre; DTA* and littermate controls. Mean ± SEM, two-way ANOVA using post-hoc Tukey's multiple comparisons test, ns: p>0.05; controls n=4, mutants n=3. **(e)** Average distance between TH^+ sympathoblasts and the border of the sympathetic chain at E10.5. Mean ± SEM, Unpaired t test ns: p=0.5153; controls n=4, mutants n=3. **(f)** Average diameter of sympathetic chain ganglia on transversal sections through E11.5 embryonic trunks at brachial and lumbar levels. Mean ± SEM, Unpaired t test, ns: p=0.999; controls n=6, mutants n=5. **(g)** Average TH^+ sympathoblast cell size. Mean ± SEM, one-way ANOVA using post-hoc Tukey's multiple comparisons test, ns: p>0.05; controls n=4, mutants n=4. **(h)** Transverse sections from thoracic (left) and lumbar (right) segments showing ectopic sympathetic differentiation along sensory nerves (TUJ1, blue) in E12 *Olig2-Cre; DTA* embryos (right) and control littermates (left). PHOX2B (red) and TH (green) are shown in magnified insets from the dotted area. Control insets display normal autonomic priming on the white ramus (left panel, arrow), but not on other peripheral nerves (right panel). Mutant insets show misplaced sympathetic clusters composed of a mixture of immature primed sympathoblasts (PHOX2-only, arrowheads) and differentiated neurons ($PHOX2B^+/TH^+$). Representative of n=4 control and n=6 mutant embryos. Scale bars: 100 µm. SG: sympathetic ganglia.

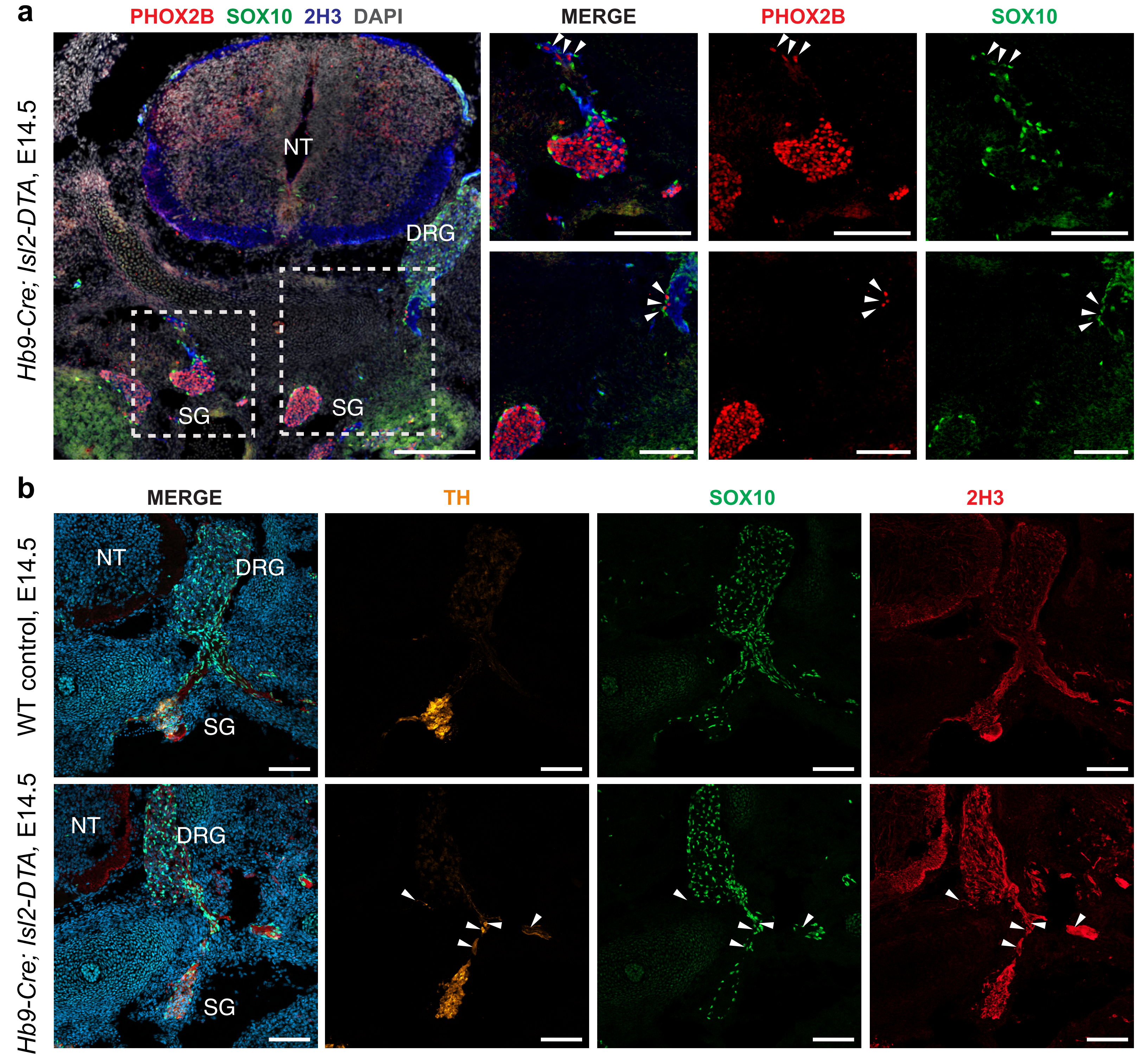
a
PHOX2B SOX10 2H3 DAPI
MERGE
PHOX2B
SOX10
Hb9-Cre; Isl2-DTA, E14.5
NT
DRG
SG
SG
b
MERGE
TH
SOX10
2H3
WT control, E14.5
NT
DRG
SG
Hb9-Cre; Isl2-DTA, E14.5
NT
DRG
SG

Figure S5: Fragmentation of sympathetic ganglia confirmed in an alternative genetic model of motor nerve ablation. Transverse sections through the trunk of E14.5 *Hb9-Cre; Isl2-DTA* embryos and controls. **(a)** Mutant embryo stained for PHOX2B (red), SOX10 (green), 2H3 (blue). Right panels are magnified from boxed areas in low magnification image (left). Arrowheads point to $SOX10^+$/$PHOX2B^+$ cells associated with sensory nerve fibers at a distance from the main sympathetic ganglia. **(b)** Sections from *Hb9-Cre; Isl2-DTA* (bottom) and control embryos (top) stained for TH (orange), SOX10 (green), 2H3 (red). Arrowheads point to ectopic $SOX10^+$/TH^+ cells associated with the sensory fibers. All images are representative of n=4 embryos. Scale bars: a: 200 µm (left), 100 µm (right); b: 100 µm. NT: neural tube, DRG: dorsal root ganglion, SG: sympathetic chain ganglia.

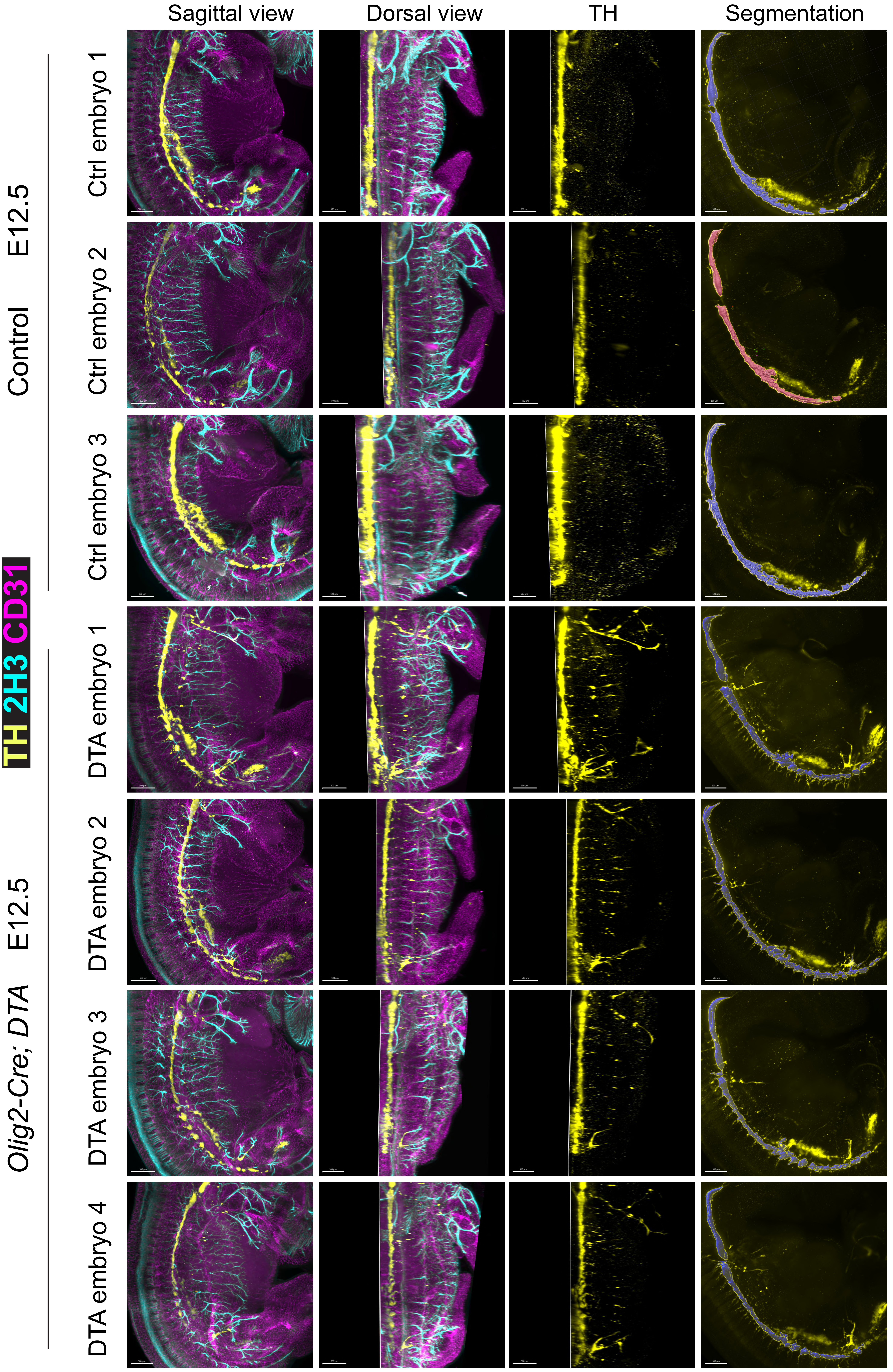

Sagittal view
Dorsal view
TH
Segmentation
E12.5
Control
Ctrl embryo 1
Ctrl embryo 2
Ctrl embryo 3
TH 2H3 CD31
DTA embryo 1
DTA embryo 2
DTA embryo 3
DTA embryo 4
E12.5
Olig2-Cre; DTA

Figure S6: Examples of whole mount immunostaining and surface segmentation with Imaris software to measure sympathetic chain volumes. Whole mount immunostaining for TH, 2H3, and CD31 identify the sympathetic chain, peripheral nerves, and blood vessels in E12.5 *Olig2-Cre; DTA* and control littermates (Cre-negative). The leftmost column shows sagittal views of embryos at the midline, and the second column shows the same z-stack after a 90-degree rotation about the anteroposterior axis. While TH staining shows some variability from embryo-to-embryo (third column), automatic thresholding within Imaris software identifies the sympathetic chain boundaries (right column). An initial mask was applied to include only the sympathetic chain and exclude other TH^+ cells (misplaced sympathoblasts, pelvic ganglion, adrenals and paraganglia). Replicates are shown. Scale bars: 500 μm.

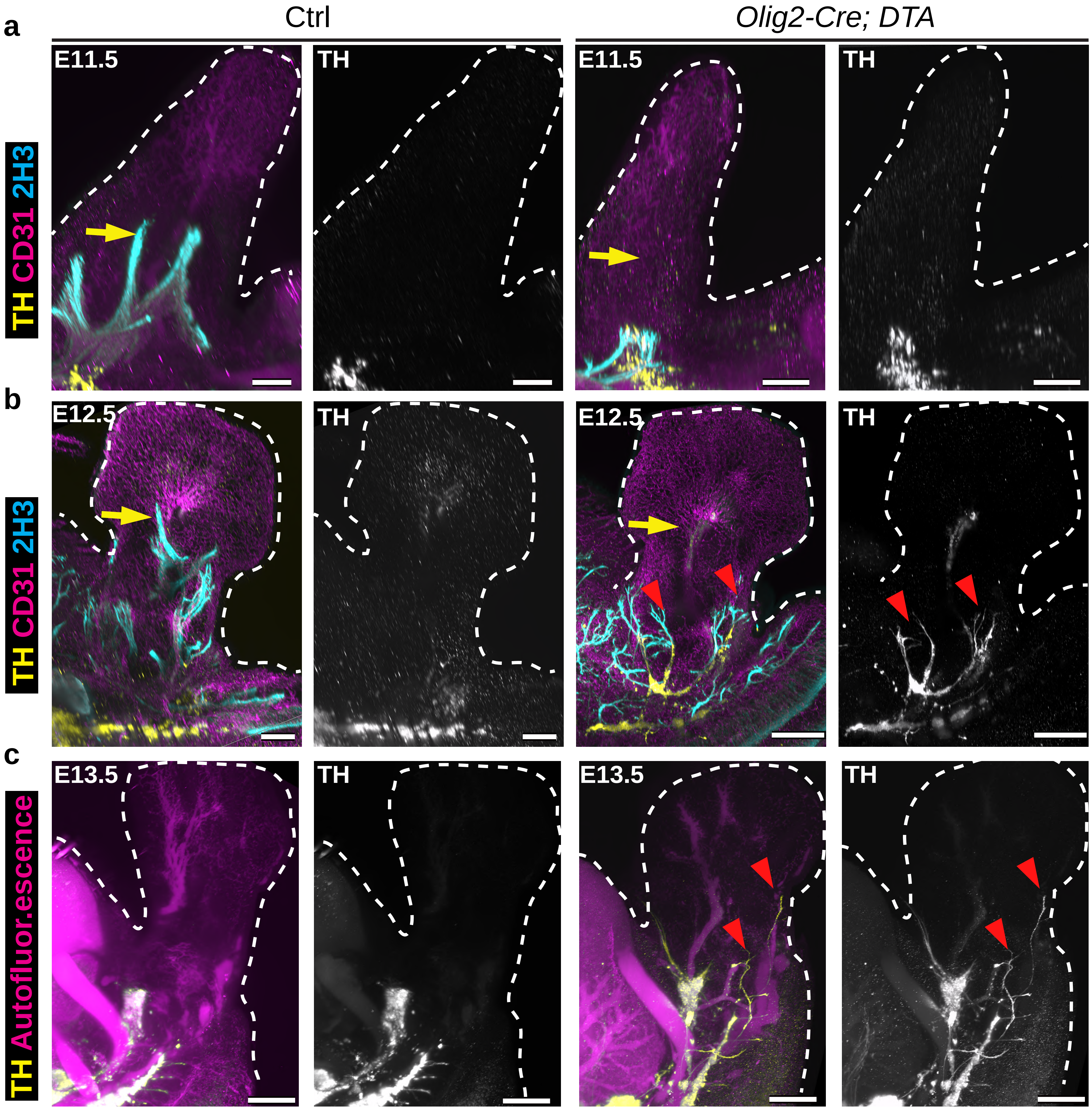
a
Ctrl
Olig2-Cre; DTA
E11.5
TH
E11.5
TH
TH CD31 2H3
b
E12.5
TH
E12.5
TH
TH CD31 2H3
c
E13.5
TH
E13.5
TH
TH Autofluor.escence

Figure S7: Motor ablation results in aberrant sympathetic innervation of the hindlimbs.
Whole mount immunostaining for TH, 2H3, CD31 to visualize peripheral nerves innervating the hindlimb region of E11.5 **(a)**, E12.5 **(b)**, or E13.5 **(c)** control (left) and *Olig2-Cre; DTA* (right) embryos from Figure 5. Dotted lines outline the hindlimb identified by autofluorescence. Red arrowheads point to inappropriate sympathetic innervation extending into the hindlimb. Note the loss of $2H3^{+}/TH^{-}$ motor nerves in the limbs of mutant embryos (yellow arrows). Representative images of at least 3 embryos per condition. Scale bars: a: 300 µm; b-c: 500 µm.

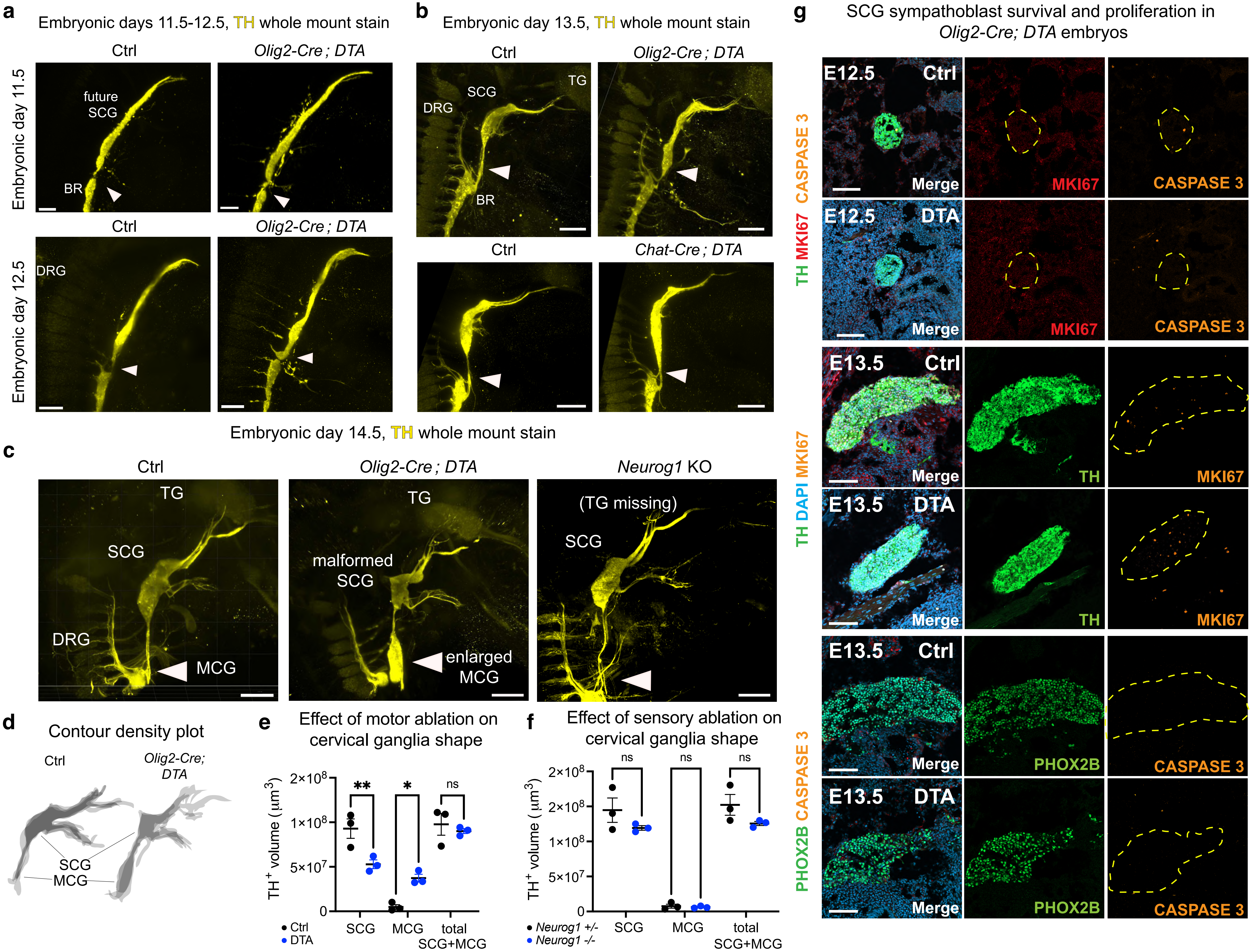

a
Embryonic days 11.5-12.5, TH whole mount stain
Ctrl
Olig2-Cre ; DTA
Embryonic day 11.5
future SCG
BR
Embryonic day 12.5
DRG
b
Embryonic day 13.5, TH whole mount stain
SCG
TG
DRG
BR
Chat-Cre ; DTA
c
Embryonic day 14.5, TH whole mount stain
Neurog1 KO
TG
SCG
malformed SCG
(TG missing)
MCG
enlarged MCG
d
Contour density plot
Olig2-Cre; DTA
SCG
MCG
e
Effect of motor ablation on cervical ganglia shape
f
Effect of sensory ablation on cervical ganglia shape
TH+ volume (µm3)
2×10^8
1×10^8
5×10^7
0
**
*
ns
Ctrl
DTA
Neurog1 +/-
Neurog1 -/-
total SCG+MCG
g
SCG sympathoblast survival and proliferation in Olig2-Cre; DTA embryos
E12.5
E13.5
Merge
MKI67
CASPASE 3
TH
PHOX2B
TH MKI67 CASPASE 3
TH DAPI MKI67
PHOX2B CASPASE 3

Figure S8: Cervical sympathetic ganglia development depends on visceromotor innervation.
(a) Sagittal view of TH whole mount immunostaining shows cervical ganglia anlagen in E11.5 and E12.5 *Olig2-Cre; DTA* (right) and control (left) embryos from Figure 5. Arrowheads point to the thinned region between cervical and brachial sympathetic ganglia in controls, which becomes progressively larger in *Olig2-Cre; DTA* embryos. Representative of 3-4 embryos per condition. **(b)** TH whole mount immunostaining in E13.5 embryos with motor ablation genotypes in right panels (*Olig2-Cre; DTA*, top and *Chat-Cre; DTA*, bottom) and corresponding littermate controls (left). Cervical ganglia morphology is severely affected (arrowheads) in *Olig2-Cre; DTA* embryos (ablation of motor neuron progenitors), while *Chat-Cre; DTA* embryos (ablation of mature motor neurons) present milder defects (arrowheads). Representative of 4-6 embryos per condition. **(c)** TH whole mount immunostaining in E14.5 controls (left), motor nerve-ablated embryos (*Olig2-Cre; DTA*, middle) and cervical sensory nerve-ablated embryos (*Neurog1 KO*, right). Medial cervical ganglion (MGC) appears enlarged in *Olig2-Cre; DTA* but not in *Neurog1 KO* and controls (arrowheads). **(d)** Contour density of cervical ganglia shaded at low opacity to show shape variation across 3 *Olig2-Cre; DTA* and 3 control littermate embryos. **(e-f)** Quantification of the superior cervical ganglia (SCG) and medial cervical ganglia (MCG) volumes in E14.5 *Olig2-Cre; DTA* embryos and controls (e), as well as *Neurog1* heterozygote and full KO (f). Each dot represents the volume of a single SCG, MCG or the combined total volume of SCG+MCG. Mean ± SEM Ordinary one-way ANOVA with Šídák's multiple comparisons test (*) p=0.0268; (**) p=0.0070; ns: p>0.05; n=3 embryos per genotype. **(g)** Transverse sections through SCG of *Olig2-Cre; DTA* and controls stained for TH, MKI67, and cleaved CASPASE3 at E12.5 (top panels). Middle and bottom panels show sagittal sections of E13.5 SCG stained for TH and MKI67 (middle) or cleaved CASPASE3 and PHOX2B (bottom). Dotted lines outline the cervical ganglia. In both mutants and controls, proliferating and apoptotic cells are rarely observed in the ganglia. Images represent n=3 embryos per genotype (top and middle panels) and n=1 embryo per genotype (bottom panels). WT: wildtype, TG: trigeminal ganglia, DRG: dorsal root ganglia, SCG: superior cervical ganglion, MCG: medial cervical ganglion, BR: brachial level. Scale bars: a: 200 µm (top) and 300 µm (bottom); b, c: 500 µm; g: 100 µm.

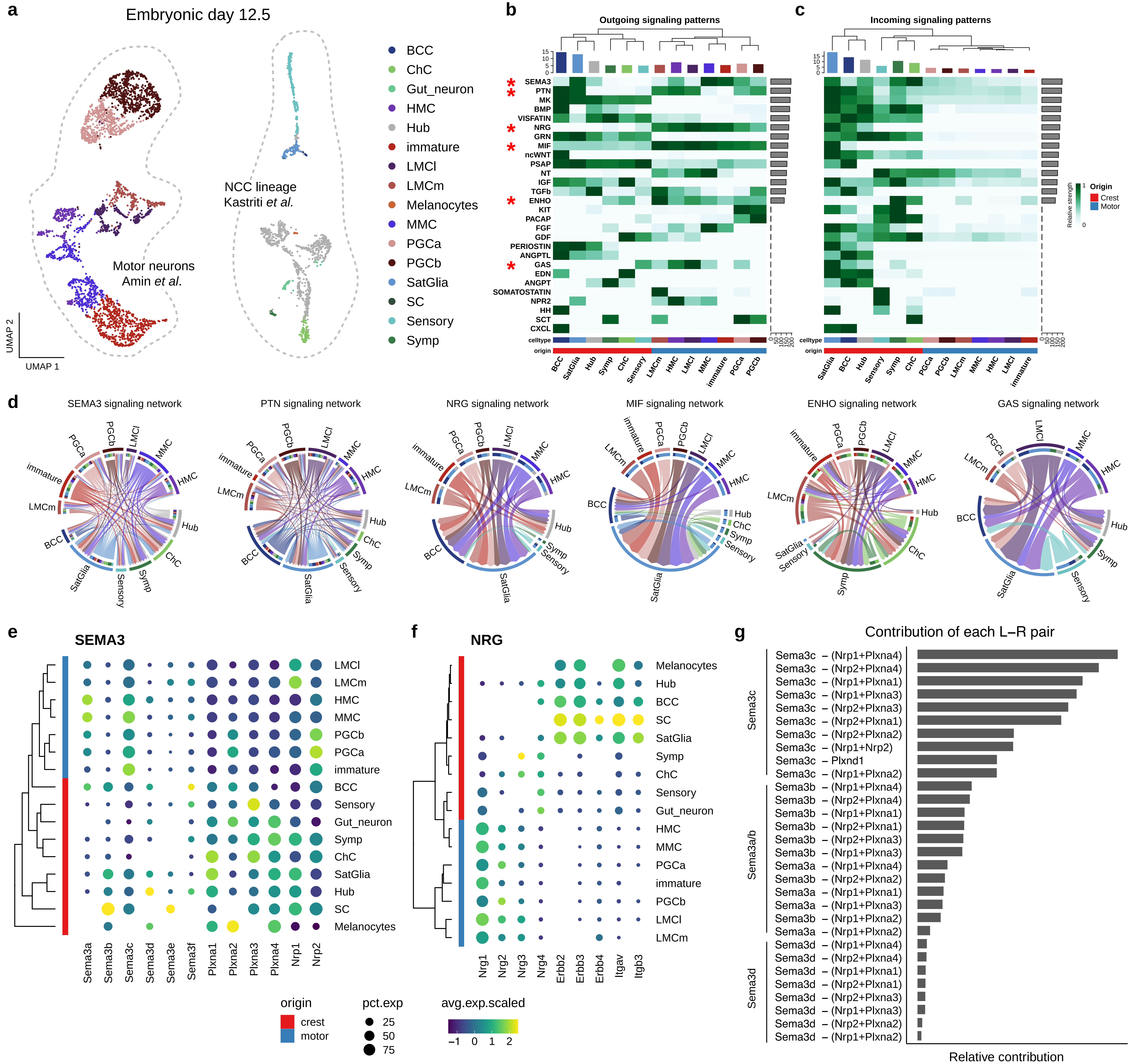

a
Embryonic day 12.5
NCC lineage Kastriti et al.
Motor neurons Amin et al.
UMAP 1
UMAP 2
BCC
ChC
Gut_neuron
HMC
Hub
immature
LMCl
LMCm
Melanocytes
MMC
PGCa
PGCb
SatGlia
SC
Sensory
Symp
b
Outgoing signaling patterns
c
Incoming signaling patterns
SEMA3
PTN
MK
BMP
VISFATIN
NRG
GRN
MIF
ncWNT
PSAP
NT
IGF
TGFb
ENHO
KIT
PACAP
FGF
GDF
PERIOSTIN
ANGPTL
GAS
EDN
ANGPT
SOMATOSTATIN
NPR2
HH
SCT
CXCL
celltype
origin
Relative strength
Origin
Crest
Motor
d
SEMA3 signaling network
PTN signaling network
NRG signaling network
MIF signaling network
ENHO signaling network
GAS signaling network
e
SEMA3
Sema3a
Sema3b
Sema3c
Sema3d
Sema3e
Sema3f
Plxna1
Plxna2
Plxna3
Plxna4
Nrp1
Nrp2
f
NRG
Nrg1
Nrg2
Nrg3
Nrg4
Erbb2
Erbb3
Erbb4
Itgav
Itgb3
origin
crest
motor
pct.exp
25
50
75
avg.exp.scaled
−1
0
1
2
g
Contribution of each L–R pair
Sema3c – (Nrp1+Plxna4)
Sema3c – (Nrp2+Plxna4)
Sema3c – (Nrp1+Plxna1)
Sema3c – (Nrp1+Plxna3)
Sema3c – (Nrp2+Plxna3)
Sema3c – (Nrp2+Plxna1)
Sema3c – (Nrp2+Plxna2)
Sema3c – (Nrp1+Nrp2)
Sema3c – Plxnd1
Sema3c – (Nrp1+Plxna2)
Sema3b – (Nrp1+Plxna4)
Sema3b – (Nrp2+Plxna4)
Sema3b – (Nrp1+Plxna1)
Sema3b – (Nrp2+Plxna1)
Sema3b – (Nrp2+Plxna3)
Sema3b – (Nrp1+Plxna3)
Sema3a – (Nrp1+Plxna4)
Sema3b – (Nrp2+Plxna2)
Sema3a – (Nrp1+Plxna1)
Sema3a – (Nrp1+Plxna3)
Sema3b – (Nrp1+Plxna2)
Sema3a – (Nrp1+Plxna2)
Sema3d – (Nrp1+Plxna4)
Sema3d – (Nrp2+Plxna4)
Sema3d – (Nrp1+Plxna1)
Sema3d – (Nrp2+Plxna1)
Sema3d – (Nrp2+Plxna3)
Sema3d – (Nrp1+Plxna3)
Sema3d – (Nrp2+Plxna2)
Sema3d – (Nrp1+Plxna2)
Sema3c
Sema3a/b
Sema3d
Relative contribution

Figure S9: Predicted molecular interactions between motor neurons and neural crest lineages.
(a) Merged UMAP embedding of single-cell transcriptomics datasets from E12.5 trunk neural crest derivatives (traced via *Sox10-Cre*^{ERT2}) and E12.5 spinal motor neurons (labeled via *Hb9-GFP*) with color-coded cell clusters annotated according to the original studies. "Hub" refers to the hub state of SCPs. **(b-c)** Heatmaps of predicted outgoing (b, ligands) and incoming (c, receptors) pathways for each cell type sorted by relative signaling strength (shown as a bar graph at the right side of each heatmap) computed by *CellChat.* The strongest inferred signals from motor neurons (blue) to neural crest cell derivatives (red) are marked by asterisks. **(d)** Inferred signaling networks showing aggregated expression of all ligand-receptor pairs pertaining to the selected candidate signaling pathways predicted by *CellChat*. Chord plots show the directionality of signaling between each cluster. **(e-f)** Dot plot showing gene expression of ligands and receptors of Semaphorin (e) and Neuregulin (f) pathways. The color scale corresponds to the average gene expression per cluster. Dot size corresponds to the proportion of cells expressing the gene in the cluster. The red/blue bars indicate dataset of origin. **(g)** Top SEMA3 receptor-ligand pairs ordered by their relative contribution to the *CellChat* prediction. MN: motor neuron; NT: neural tube; NCC: neural crest cell; BCC: boundary cap; ChC: chromaffin cells; HMC: hypaxial motor column; Hub: Schwann cell precursors; LMCl: lateral motor column lateral division; LMCm: LMC medial division; MMC: medial motor column; PGCa/b: preganglionic motor column (a/b) divisions; SatGlia: satellite glia; SC: Schwann Cells; Symp: sympathetic neurons.

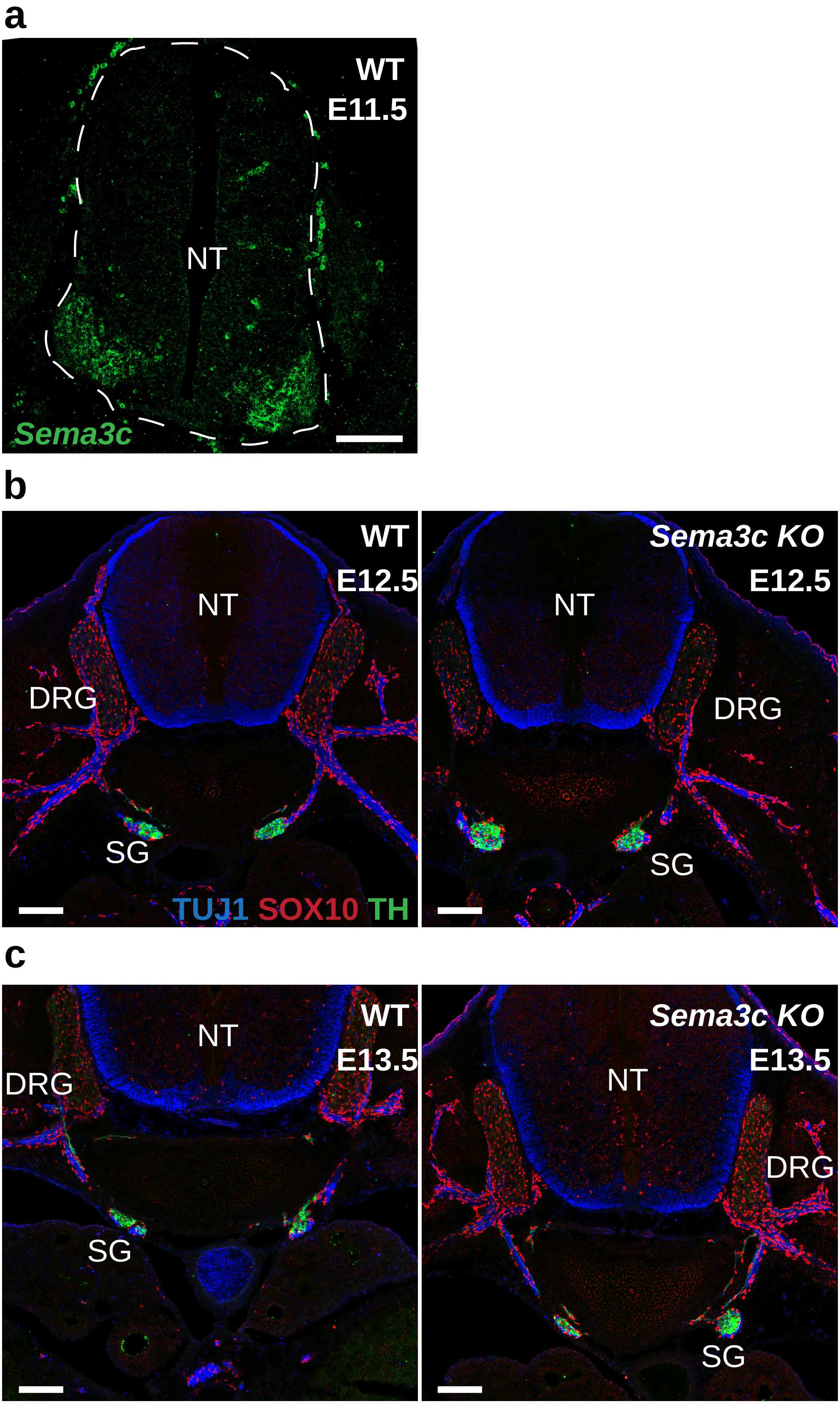
a
WT
E11.5
NT
Sema3c
b
WT
E12.5
NT
DRG
SG
TUJ1 SOX10 TH
Sema3c KO
E12.5
NT
DRG
SG
c
WT
E13.5
NT
DRG
SG
Sema3c KO
E13.5
NT
DRG
SG

Figure S10: Semaphorin 3C does not influence sympathoblast positioning. (a) *In situ* hybridization chain reaction (HCR) of Semaphorin 3C (Sema3C) in transverse sections of E11.5 embryo neural tube (outlined, n=2 embryos). **(b-c)** Trunk transverse sections of E12.5 (b) and E13.5 (c) *Sema3C KO* embryos (right) and controls (left) stained for TH (green), SOX10 (red), and TUJ1 (blue) to mark sympathetic neurons, SCPs/glia, and peripheral nerves, respectively. *Sema3C KO* embryos do not display apparent defects in sympathetic nervous system assembly. Representative images of n=3 E12.5 embryos per genotype and n=4 E13.5 embryos per genotype. DRG: dorsal root ganglia; SG: sympathetic chain ganglia; NT: neural tube. Scale bars: 100 µm.

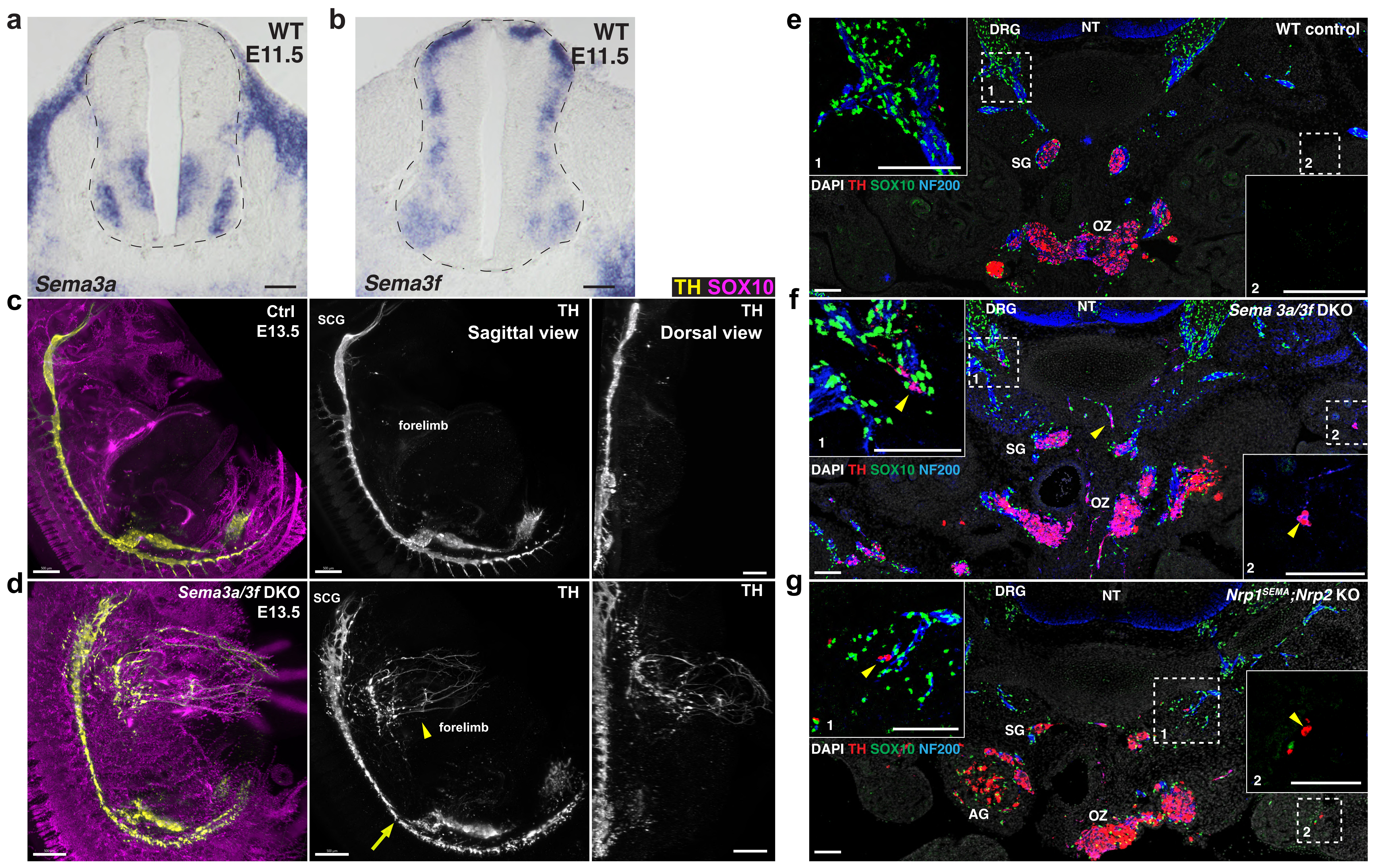

a
WT
E11.5
Sema3a
b
WT
E11.5
Sema3f
TH SOX10
c
Ctrl
E13.5
SCG
TH
Sagittal view
forelimb
TH
Dorsal view
d
Sema3a/3f DKO
E13.5
SCG
TH
forelimb
TH
e
DRG
NT
WT control
1
SG
2
DAPI TH SOX10 NF200
OZ
f
DRG
NT
Sema 3a/3f DKO
1
SG
2
DAPI TH SOX10 NF200
OZ
g
DRG
NT
Nrp1SEMA;Nrp2 KO
1
SG
DAPI TH SOX10 NF200
AG
OZ
2

Figure S11: Sema3 signaling regulates sympathoblast positioning. (a-b) *In situ* hybridization for *Sema3a* (a) and *Sema3f* (b) in transverse sections of the embryonic neural tube at E11.5. Representative of 6 embryos. **(c-d)** Whole mount immunostaining of sympathetic ganglia (TH, yellow, and SOX10, magenta) in control (c) and *Sema3a/Sema3f* double KO embryos (d) at E13.5. TH is shown separately in sagittal (middle) and dorsal (right) views to highlight aberrant innervation of forelimbs (arrowheads) and morphological defects (arrow) in mutants. Images representative of n=3 control embryos and n=1 Sema3a/Sema3f double KO embryo. **(e-g)** Transverse lumbar sections of E13.5 control (e), *Sema3a/Sema3f* double KO (f) and $Nrp1^{SEMA/SEMA}$; $Nrp2^{-/-}$ (g) embryos. Immunofluorescence for TH (red), SOX10 (green), NF200 (blue) and DAPI (grey) identifies sympathetic neurons, SCPs/glia, peripheral nerves and nuclei, respectively. Insets are magnifications of the boxed areas (without DAPI staining). Yellow arrowheads mark misplaced sympathoblasts. The confocal immunohistochemistry images are representative of n=6 control embryos, n=4 $Nrp1^{SEMA/SEMA}$; $Nrp2^{-/-}$ embryos, and n=2 *Sema3a/Sema3f* double KO embryos between stages E12.5-13.5. DRG: dorsal root ganglia; SG: sympathetic chain ganglia; NT: neural tube; OZ: organ of Zuckerkandl; AG; adrenal gland; SCG: superior cervical ganglion. Scale bars: a, b: 100 µm; c, d: 0.5mm; e, f, g: 20 µm.

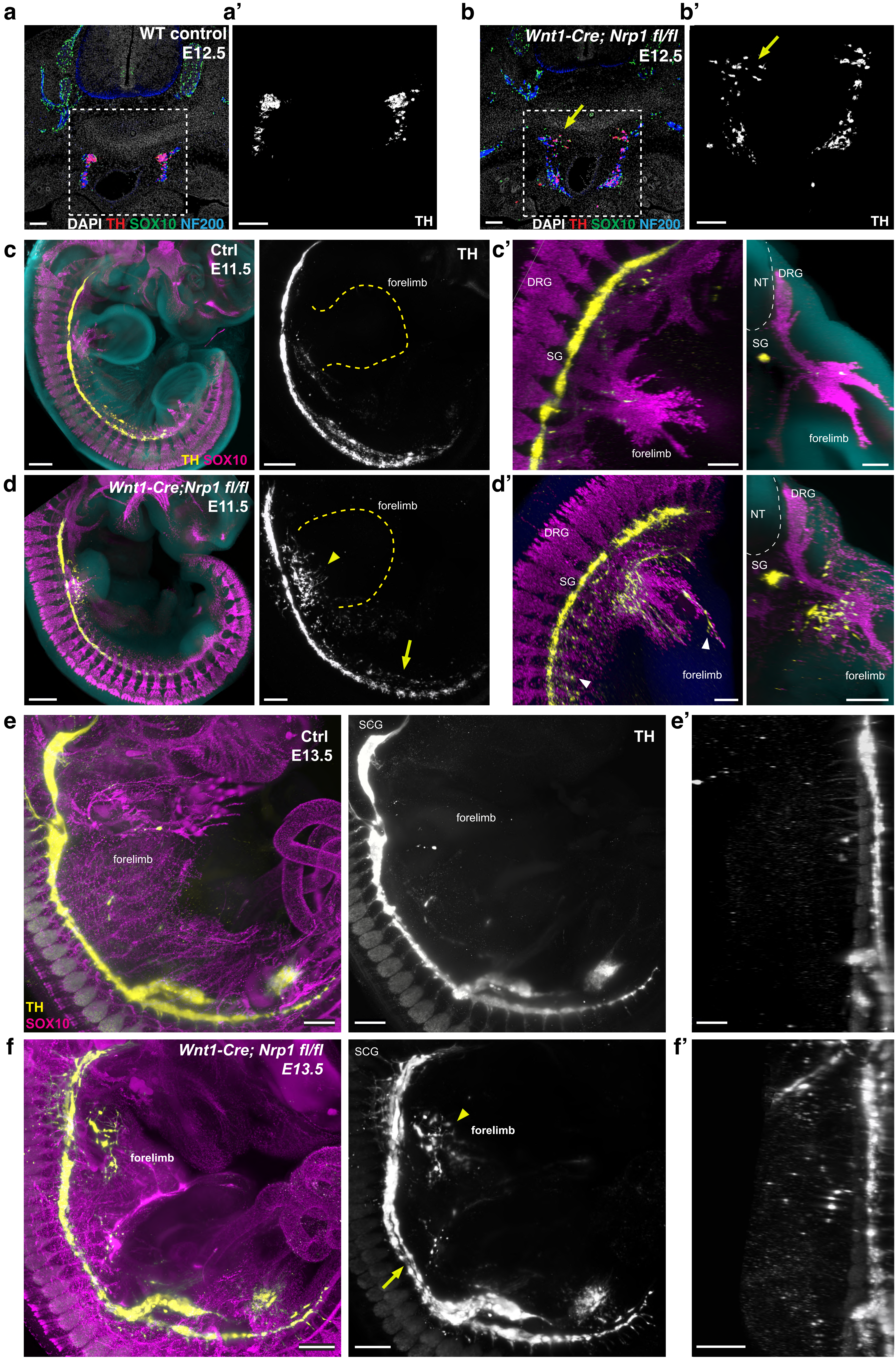

a
WT control
E12.5
DAPI TH SOX10 NF200
a'
TH
b
Wnt1-Cre; Nrp1 fl/fl
E12.5
DAPI TH SOX10 NF200
b'
TH
c
Ctrl
E11.5
TH SOX10
TH
forelimb
c'
DRG
SG
forelimb
NT
DRG
SG
forelimb
d
Wnt1-Cre;Nrp1 fl/fl
E11.5
forelimb
d'
DRG
SG
forelimb
NT
DRG
SG
forelimb
e
Ctrl
E13.5
forelimb
TH
SOX10
SCG
TH
forelimb
e'
f
Wnt1-Cre; Nrp1 fl/fl
E13.5
forelimb
SCG
forelimb
f'

Figure S12: Neuropilin 1 in the neural crest lineage regulates sympathoblast positioning. **(a-b)** Immunofluorescence staining of TH (red), SOX10 (green), NF200 (blue) and DAPI (grey) to mark sympathetic neurons, glial cells, peripheral nerves and nuclei, respectively, in controls (a) and *Wnt1-Cre; Nrp1$^{flox/flox}$* (b) embryos. TH is shown separately in a' and b' (magnifications of the boxed areas in a and b). Arrow points to fragmented sympathetic ganglia in mutants. Images are representative of at least 3 embryos per genotype. **(c-f)** Whole mount immunofluorescence of E11.5 (c, d) and E13.5 (e, f) control (c, e) and *Wnt1-Cre; Nrp1$^{flox/flox}$* (d, f) embryos stained for TH (yellow) and SOX10 (magenta). Nuclei are visualized by Hoechst staining (cyan). The forelimbs are outlined. (c') and (d') show magnification of whole mount (left) and transverse view (right) of forelimbs from c and d, respectively. (e') and (f') show the dorsal view. Yellow arrowheads mark misplaced sympathoblasts in the forelimbs, while arrows point to morphological defects of the sympathetic chain in mutants. Images are representative of n=4 E11.5 control embryos, n=5 E13.5 control embryos, n=3 E11.5 mutant embryos, and n=2 E13.5 mutant embryos. DRG: dorsal root ganglia; SG: sympathetic chain ganglia; NT: neural tube; SCG: superior cervical ganglion. Scale bars a, b: 20 µm; c-d: 500 µm; c': 200 µm; d': 300 µm; e, f: 500 µm; e', f': 500 µm.
