# Supplementary material for: Motor innervation directs the correct development of the mouse sympathetic nervous system
Source: Nat Commun. 2024 Aug 16;15:7065. doi: 10.1038/s41467-024-51290-0 (PMC11329663; doi:10.1038/s41467-024-51290-0)
Supplement: Supplementary file 3 — Reporting Summary [file 41467_2024_51290_MOESM3_ESM.pdf]

nature portfolio

Corresponding author(s): Igor Adameyko

Last updated by author(s): Jul 15, 2024

Reporting Summary

Nature Portfolio wishes to improve the reproducibility of the work that we publish. This form provides structure for consistency and transparency in reporting. For further information on Nature Portfolio policies, see our Editorial Policies and the Editorial Policy Checklist.

Statistics

For all statistical analyses, confirm that the following items are present in the figure legend, table legend, main text, or Methods section.

n/a	Confirmed	
☐	☒	The exact sample size (*n*) for each experimental group/condition, given as a discrete number and unit of measurement
☐	☒	A statement on whether measurements were taken from distinct samples or whether the same sample was measured repeatedly
☐	☒	The statistical test(s) used AND whether they are one- or two-sided<br>*Only common tests should be described solely by name; describe more complex techniques in the Methods section.*
☐	☒	A description of all covariates tested
☐	☒	A description of any assumptions or corrections, such as tests of normality and adjustment for multiple comparisons
☐	☒	A full description of the statistical parameters including central tendency (e.g. means) or other basic estimates (e.g. regression coefficient) AND variation (e.g. standard deviation) or associated estimates of uncertainty (e.g. confidence intervals)
☐	☒	For null hypothesis testing, the test statistic (e.g. *F*, *t*, *r*) with confidence intervals, effect sizes, degrees of freedom and *P* value noted<br>*Give P values as exact values whenever suitable.*
☒	☐	For Bayesian analysis, information on the choice of priors and Markov chain Monte Carlo settings
☒	☐	For hierarchical and complex designs, identification of the appropriate level for tests and full reporting of outcomes
☒	☐	Estimates of effect sizes (e.g. Cohen's *d*, Pearson's *r*), indicating how they were calculated

Our web collection on statistics for biologists contains articles on many of the points above.

Software and code

Policy information about availability of computer code

Data collection	ZEISS Zen Blue software was used to acquire the confocal microscopy images. Light sheet images were acquired in the .czi format in Zen (Black edition, version 3)
Data analysis	10x Genomics Cell Ranger v7.0.0 [65] was used to process raw sequencing data. This pipeline converted Illumina base call files into Fastq format, aligned sequencing reads to a mm39 transcriptome using the STAR aligner [66], and quantified the expression of transcripts in each cell using Chromium barcodes. The Cell Ranger outputs were given to the velocyto.py pipeline (version 0.17.17) [67] to generate spliced/unspliced expression matrices further used for RNA velocity estimation. Scanpy package pipeline (version 1.9.3) [68] was used for the downstream analysis. To retain only high-quality cells, we filtered out the cells with high mitochondrial content (more than 10%); the cells with less than 2000 UMIs (1000 UMIs for Egr2_E12 dataset); and cells defined as putative doublets (with a doublet score equal to or greater than 0.2, calculated by Scrublet version 0.2.3) [69]. The filtered datasets first were analyzed separately to extract the cells belonging to BCC lineage and then integrated with Harmony (3000 highly variable genes, 30 principal components (PC), max number of iterations = 20) [70]. The new PCs adjusted by Harmony were used to compute a nearest neighbor graph with further clustering and embedding by the Leiden algorithm [71] and UMAP (Uniform Manifold Approximation and Projection), respectively. Cell types were identified based on the Leiden clusters and marker gene expression. For RNA velocity estimation, we used the scvelo package (version 0.3.1) [72] with a dynamical model to learn the transcriptional dynamics of splicing kinetics.<br><br>Single cell transcriptomics datasets used for the study were downloaded from (https://github.com/LouisFaure/glialfates_paper) and ArrayExpress accession: E-MTAB-10571. Standard pre-processing workflow such as QC, cell selection, data normalization, identification of highly variable features, scaling, dimensional reduction, clustering, and integration of the datasets was performed in R (version 4.3.2) using Seurat (version 5.0.3) according to the instructions provided (https://satijalab.org). Cell interaction analysis was performed using the R package CellChat (v 1.6.1).

Volumetric quantification of pelvic ganglia was performed using Imaris (version 9.5, Bitplane) with the surface generation tool and a fixed intensity threshold for all samples. Volumetric quantification of sympathetic chain volumes was performed using Imaris (version 9.5, Bitplane) using the surface generating tool.

Quantification of lineage tracing experiments was performed using FIJI (ImageJ, version 2.14.0).

Light sheet images acquired in the .czi format in Zen (Black edition, version 3) were processed by stitching with Arivis Vision 4D (Zeiss, version 4.0), down-sampling (1:2 in the XY plane) in FIJI (ImageJ, version 2.14.0), conversion to .ims files using Imaris File Converter (Bitplane, version 9.5), and downstream analysis with Imaris (Bitplane, version 9.5).

Statistical analysis was performed with GraphPad Prism (version 9.5.1) software

The code used for single cell analysis, both tracing boundary cap cells and for predicting cell interactions between motor neurons and SCPs, can be found at the GitHub link: https://github.com/ipoverennaya/motor_nerve_paper.

For manuscripts utilizing custom algorithms or software that are central to the research but not yet described in published literature, software must be made available to editors and reviewers. We strongly encourage code deposition in a community repository (e.g. GitHub). See the Nature Portfolio guidelines for submitting code & software for further information.

Data

Policy information about availability of data

All manuscripts must include a data availability statement. This statement should provide the following information, where applicable:

- Accession codes, unique identifiers, or web links for publicly available datasets
- A description of any restrictions on data availability
- For clinical datasets or third party data, please ensure that the statement adheres to our policy

Source data are provided with this paper as a Source Data File. Information provided in the text, figures and supplementary information contained in the present manuscript is sufficient to assess whether the claims of this study are supported by the evidence. Neural crest single cell transcriptomics datasets used for the cell interaction study are available from GEO via accession GSE201257 (https://www.ncbi.nlm.nih.gov/geo/query/acc.cgi?acc=GSE201257) and are viewable from https://adameykolab.hifo.meduniwien.ac.at/cellxgene_public/. Motor neuron datasets can be found using the ArrayExpress accession: E-MTAB-10571 (https://www.ebi.ac.uk/biostudies/arrayexpress/studies/E-MTAB-10571). Dataset for the lineage tracing study has been submitted to GEO under accession code GSE261748 (NCBI tracking system #24559159). Raw microscopy files generated for this research project are available to interested parties upon request.

Human research participants

Policy information about studies involving human research participants and Sex and Gender in Research.

Reporting on sex and gender	no human subjects were studied
Population characteristics	see above
Recruitment	no human subjects were studied
Ethics oversight	*Identify the organization(s) that approved the study protocol.*

Note that full information on the approval of the study protocol must also be provided in the manuscript.

Field-specific reporting

Please select the one below that is the best fit for your research. If you are not sure, read the appropriate sections before making your selection.

☒ Life sciences ☐ Behavioural & social sciences ☐ Ecological, evolutionary & environmental sciences

For a reference copy of the document with all sections, see nature.com/documents/nr-reporting-summary-flat.pdf

Life sciences study design

All studies must disclose on these points even when the disclosure is negative.

Sample size	No statistical method was used to predetermine sample size.
Data exclusions	Data was not excluded from the study.
Replication	The efficacy of our motor nerve ablation model, and the impact it has on the sympathetic ganglia development, is reproducible because multiple collaborators on this study, situated in different countries (Italy, Sweden, Vienna, Australia), have found the same phenotype using the same or comparable mouse strains (Hb9;Isl2DTA, or Olig2;R26DTA).

Randomization	The experiments were not randomized.
Blinding	The investigators were not blinded to allocation during experiments and outcome assessment.

Reporting for specific materials, systems and methods

We require information from authors about some types of materials, experimental systems and methods used in many studies. Here, indicate whether each material, system or method listed is relevant to your study. If you are not sure if a list item applies to your research, read the appropriate section before selecting a response.

Materials & experimental systems

n/a	Involved in the study
☐	☒ Antibodies
☒	☐ Eukaryotic cell lines
☒	☐ Palaeontology and archaeology
☐	☒ Animals and other organisms
☒	☐ Clinical data
☒	☐ Dual use research of concern

Methods

n/a	Involved in the study
☒	☐ ChIP-seq
☒	☐ Flow cytometry
☒	☐ MRI-based neuroimaging

Antibodies

Antibodies used

Rabbit polyclonal anti-TH (1:800, Pel-Freez Biologicals, #P40101-150, RRID:AB_2617184), sheep polyclonal anti–TH (1:2000, Novus Biologicals, #NB300-110), chicken polyclonal anti-TH (1:500, Abcam, #ab76442, RRID:AB_1524535), rabbit polyclonal anti-Hb9 (1:8000, gift from Samuel Pfaff's laboratory [63]), mouse monoclonal anti-bIII tubulin/TUJ1 (1:500, Promega, #G712A), mouse monoclonal anti-bIII tubulin/TUJ1 (1:1000, Abcam #ab7751, clone TU-20, RRID:AB_306045), rabbit polyclonal anti-bIII tubulin/TUJ1 (1:1000, Synaptic Systems, cat#302302, RRID:AB_10637424), chicken polyclonal anti-GFP (1:500, Aves Labs Inc., #GFP-1020, RRID:AB_10000240), chicken polyclonal anti-GFP (1:1000, Abcam, ab13970, polyclonal, RRID:AB_300798), rabbit polyclonal anti-GFP (1:5000, Thermo/LifeTech, #A6455, lot#2126798, RRID:AB_221570), goat polyclonal anti-PHOX2B (R&D, 1:1000, #AF4940), mouse monoclonal anti-Neurofilament/NF200 (1:200, Developmental Hybridoma Studies Bank, clone 2H3), goat polyclonal anti-SOX10 (1:500, Santa-Cruz, #sc-17342), rabbit monoclonal anti-SOX10 (1:2000, Abcam, # ab155279, clone EPR4007, RRID:AB_2650603), goat anti-human SOX10 (1:800, R&D Systems, #AF2864, RRID:AB_442208), rabbit polyclonal anti-PRPH (1:500, Chemicon, #AB1530, RRID:AB_90725), rabbit monoclonal anti-KI67 (1:500, Thermo Scientific, #RM-9106, clone SP6, RRID:AB_2341197), rat monoclonal anti-PECAM (1:300, BD Pharmingen, #553370, RRID:AB_394816), goat polyclonal anti-PECAM (1:300, R&D systems #AF3628, RRID:AB_2161028), rabbit monoclonal anti-ITGA4/CD49d (1:500, Invitrogen, #MA5-27947, clone RM268, RRID:AB_2744984), rabbit monoclonal anti-Cleaved Caspase 3/Asp175 (1:500, Cell signaling #96645, clone 5A1E), rabbit polyclonal anti-TrkA (1:500, # 06-574 Sigma-Aldrich, RRID:AB_310180). DAPI (Thermo Fisher Scientific, 1:10,000, #D1306) was used concomitantly with secondary antibodies diluted in PBST buffer. For detection of the primary antibodies, secondary antibodies raised in donkey and conjugated with Alexa-405, -488, -555 and -647 fluorophores were used (1:1000, Molecular Probes, Thermo Fisher Scientific).

Validation

All antibodies used are validated by the fact that they stain the expected cell populations during development. Most antibodies we used are standard in the field and have been cited numerous times, and validated by the respective manufacturers (see RRID for each antibody).

rabbit anti-TH (1:1000, Pel-Freez Biologicals, #P40101-150) - Species Reactivity: All mammalian and at least some non-mammalian forms of the enzyme in Western blots and in IHC/IF.

sheep anti–TH (1:2000, Novus Biologicals, #NB300-110) - The antibody recognizes all mammalian and at least some non-mammalian forms of the enzyme in Western blot and in IHC/IF. Amphibian reactivity reported in scientific literature (PMID: 28867550).

chicken anti- GFP (1:500, Aves Labs Inc., #GFP-1020) - Antibodies were analyzed by western blot analysis (1:5000 dilution) and immunohistochemistry (1:500 dilution) using transgenic mice expressing the GFP gene product. Western blots were performed using BlokHen® (Aves Labs) as the blocking reagent, and HRP-labeled goat anti-chicken antibodies (Aves Labs, Cat. #H-1004) as the detection reagent. Immunohistochemistry used tetramethyl rhodamine-labeled anti-chicken IgY.

goat anti-human SOX10 (1:800, R&D Systems, #AF2864) - Detects human SOX10 in direct ELISAs and Western blots and mouse SOX10 in IHC on cryosections as shown in multiple publications (PMID: 28684471, PMCID: PMC6355685 and etc).

Animals and other research organisms

Policy information about studies involving animals; ARRIVE guidelines recommended for reporting animal research, and Sex and Gender in Research

Laboratory animals

Mice were kept in standard conditions: 24˚C; 12h-12h light dark cycle; 40–60% humidity; food and water ad libitum. R26-Tomato

	mice were ordered from The Jackson Laboratory (stock number 007914). Plp1-CreERT2 mice were received from U. Suter laboratory (ETH Zurich, Switzerland) (http://www.informatics.jax.org/allele/MGI:2663093). R26-YFP mice were received from The Jackson Laboratory (stock number 006148, full strain name B6.129X1-Gt(ROSA)26Sortm1(EYFP)Cos/J). Hb9-Cre (also known as Mnx1-Cre) mice were received from The Jackson Laboratory, stock number 006600 (full strain name B6.129S1-Mnx1tm4(cre)Tmj/J). Isl2-DTA mice were received from The Jackson Laboratory, stock number 007942 (full strain name B6.Cg-Isl2tm1Arbr/J). R26-DTA alleles were received from The Jackson Laboratory, stock numbers 006331 (full strain name Gt(ROSA)26Sortm1(DTA)Jpmb/J) and 010527 (full strain name B6;129-Gt(ROSA)26Sortm1(DTA)Mrc/J) [58]. Chat-Cre mice were received from K. Meletis lab (Karolinska Institutet) also available from the Jackson Laboratory, stock number 006410 (full strain name B6;129S6-Chattm2(cre)Lowl/J). Olig2-Cre mouse line (C57BL6/n background) was donated by T. Jessel laboratory (Columbia University, New York, US)[59]. Hb9-GFP and MN(218-2)-GFP mouse lines (C57BL6/n background) were donated by S. Pfaff laboratory (Salk Institute, San Diego, US) [60, 61]. Mouse mutants deficient in semaphorin and neuropilin signaling (Wnt1-Cre Nrp1flox/flox, Sema3a/3f-DKO , Nrp1SEMA Nrp2 KO) as well as strains used for boundary cap cell tracing (Egr2-Cre, Prss56-Cre) have been described previously [22, 28-31, 34]. Sema3C KO mouse line (CD1 background) was donated by J. Raper (University of Pennsylvania, Philadelphia, US) and S. Chauvet Aix-Marseille Université, Marseille, France) [62].
Wild animals	no wild animals
Reporting on sex	sex/gender is not relevant to the study, because we are dealing mostly with embryonic development of sympathetic ganglia which is not different across the sexes at the stages investigated.
Field-collected samples	n/a
Ethics oversight	All animal work was permitted by the Ethical Committee on Animal Experiments (Stockholm North committee) and Animal Research Committee of IRCCS San Raffaele Hospital, and conducted in compliance with The Swedish Animal Agency's Provisions and Guidelines for Animal Experimentation recommendations under I.A.'s ethical protocol #15907-19; 18314-21 and The Italian Ministry of Health under D.B.'s protocols #1131/2016-PR and 668/2022-PR.

Note that full information on the approval of the study protocol must also be provided in the manuscript.
